# Clearing the air: Which pollution source matters most for health?

**DOI:** 10.17269/s41997-025-01142-1

**Published:** 2025-12-09

**Authors:** Ying Liu, Marianne Hatzopoulou, Stéphane Buteau, Shayamilla Mahagammulla Gamage, Sara Torbatian, Arman Ganji, Audrey Smargiassi

**Affiliations:** 1 School of Public Health, University of Montreal, Montreal, QC Canada; 2 Department of Civil and Mineral Engineering, University of Toronto, Toronto, ON Canada; 3 Center for Public Health Research (CReSP), University of Montreal, CIUSSS du Centre-Sud-de-L’Île-de-Montréal, Montreal, QC Canada

**Keywords:** PM_2.5_, NO_2_, O_3_, Intervention scenario, AQBAT, Mortality, Polair3D, PM_2.5_, NO_2_, O_3_, Intervention scenario, AQBAT (OEBQA), Mortalité, Polair3D

## Abstract

**Objectives:**

Air pollution remains a significant public health challenge, contributing to substantial morbidity and mortality. The aim of this study is to identify effective intervention strategies for mitigating air pollution and its health effects in southern Quebec.

**Methods:**

We employ the Polair3D chemical transport model to estimate population-weighted concentrations of fine particulate matter (PM_2.5_), nitrogen dioxide (NO_2_), and ozone (O_3_) at the census division level under four scenarios: replacing residential wood stoves with U.S. Environmental Protection Agency (EPA)–certified models (EPA), eliminating industrial emissions (IND), full vehicle electrification (EV), and removing emissions from refineries and smelters (RS). Health impacts were quantified with the Air Quality Benefits Assessment Tool for chronic PM_2.5_ exposure and chronic NO_2_ exposure, in addition to the commonly assessed acute NO_2_ exposure and acute O_3_ exposure.

**Results:**

All scenarios reduced air pollutant concentrations and associated mortality to varying degrees. The EPA and EV interventions are the most effective in reducing mortality, lowering deaths attributable to pollutants by 15.26% (789 deaths from 5169 to 4380) and 16.13% (834 deaths from 5169 to 4335), respectively. The EPA scenario yields the greatest reduction in PM_2.5_-related mortality, while the EV scenario provides the most benefit for NO_2_-related mortality.

**Conclusion:**

Targeted interventions can significantly reduce air pollution-related mortality. Replacing residential wood stoves and fully electrifying vehicles are particularly effective, with distinct benefits for PM_2.5_- and chronic NO_2_-related health outcomes. A multi-sectoral approach is essential to maximize public health gains.

**Supplementary Information:**

The online version contains supplementary material available at https://doi.org/10.17269/s41997-025-01142-1.

## Résumé

**Objectif:**

La pollution de l’air est un problème de santé publique, contribuant de façon substantielle à la morbidité et à la mortalité. Cette étude vise à identifier les interventions les plus efficaces pour réduire la pollution de l’air et ses impacts sanitaires dans le sud du Québec.

**Méthodes:**

Nous avons utilité le modèle “chimie-transport” Polair3D pour estimer les concentrations de particule fines (PM_2.5_), de dioxyde d’azote (NO_2_) et d’ozone (O_3_) des divisions de recensement, pondérées pour la population, selon quatre scénarios: le remplacement des appareils de combustion résidentielle du bois pour des appareils rencontrant la certification du U.S. Environmental Protection Agency (EPA)-(scénario EPA), l’élimination des émissions industrielles (IND), l’électrification des véhicules routiers (EV), et l’élimination des émissions de raffineries et fonderies (RF). Les impacts sanitaires ont été quantifiés avec l’outil AQBAT (OEBQA) pour l’exposition chronique aux PM_2.5_; les impacts de l’exposition chronique au NO_2_ a également été estimée, en plus de ceux de l’exposition aigüe au NO_2_ et à l’O_3_, généralement considérés.

**Résultats:**

Tous les scenarios réduisent les concentrations et la mortalité mais de façon variable. Les scenarios EPA et EV ont le plus d’impacts sur la mortalité, réduisant les décès attribuables aux polluants de 15.26% (789 décès, variant de 5,169 à 4,380) et 16.13% (834 décès, variant de 5,169 à 4,335), respectivement. La réduction des décès du scenario EPA est associée au contrôle des PM2.5 tandis que celle du scénario EV est associée au contrôle du NO_2_.

**Conclusion:**

Des interventions ciblées peuvent réduire la mortalité attribuable à la pollution de l’air. Le remplacement des appareils de combustion résidentielle du bois et l’électrification des véhicules sont particulièrement efficaces pour leurs bénéfices respectifs en termes de réduction des décès attribuables à l’exposition chronique aux PM_2.5_-et au NO_2_. Une approche multi-secteur est donc essentielle pour améliorer la santé publique.

## Introduction

Exposure to air pollution is widely recognized as a significant global health concern, with extensive research establishing its links to adverse health outcomes (GBD, 2020; Lee et al., 2020; Manisalidis et al., 2020). In Canada, Health Canada (2021) estimates that air pollution contributes to approximately 15,300 premature deaths annually, and it is expected to rise with population growth and aging. These health impacts not only compromise public health but also impose substantial economic burdens. To effectively reduce this burden, it is critical to understand the sources of air pollution and their respective contributions to health outcomes. Such knowledge provides essential insights for designing and implementing targeted intervention strategies aimed at reducing pollutant levels and mitigating the associated health and economic impacts.

Ambient fine particulate matter with median aerosol dynamic diameters of 2.5 µm (PM_2.5_), nitrogen dioxide (NO_2_), and ground-level ozone (O_3_) are the main pollutants associated with health effects (Health Canada, 2021). Major sources of these pollutants include residential energy combustion, transportation fuel use, industrial emissions, and energy production (Holman, 1999; Relvas et al., 2022). Common strategies for mitigating these emissions include industrial emission standards, replacing residential stoves, banning coal and wood burning, and promoting public transportation and clean fuel alternatives (Burns et al., 2020).

Several studies have modeled intervention scenarios to assess the potential for reducing air pollutant levels and their associated health benefits. Various chemical transport models, including the Community Multiscale Air Quality (CMAQ) model, the Goddard Earth Observing System chemical transport (GEOS-Chem) model, and the Weather Research Forecasting chemical transport (WRF-Chem) model, have been employed at different spatial resolutions (ranging from 12 to 50 km) to estimate air pollution concentrations under different emission reduction strategies. To quantify the potential health impacts, studies have typically applied concentration–response functions (CRFs). Johnson et al. (2020) found that reducing emissions from residential cooking, energy generation, and transportation in New York City could prevent 160–390 premature deaths attributable to PM_2.5_ exposure. Similarly, Zhou et al. (2010) and Hou et al. (2015) highlighted the substantial health benefits of reducing PM_2.5_ and O_3_ emissions from power plants and industrial sectors in China and the United States, respectively. In India, Chatterjee et al. (2023) and Upadhyay et al. (2018) identified residential combustion and industrial emissions as the largest contributors to PM_2.5_-related mortality. Likewise, Zheng et al. (2024) and Gu et al. (2024) found that industrial and residential emissions were the dominant sources of PM_2.5_ and O_3_-related health impacts in China and Southeast Asia. Liu et al. (2024) emphasized the health benefits of transitioning from coal to gas for residential heating, while Silva et al. (2016) identified the residential and commercial sectors as major contributors to global PM_2.5_ and O_3_-related mortality.

In the Canadian context, Health Canada (2023) assessed air quality and its associated health impacts across 21 sectors to identify effective mitigation strategies. The analysis revealed that residential, industrial, and transportation sectors were the leading contributors to mortality linked to chronic exposure to PM_2.5_ and acute exposure to NO₂ and O₃. These sectors collectively accounted for approximately 6500 premature deaths, representing 94.2% of the total premature deaths attributed to the 21 sectors analyzed. Notably, Quebec bore a disproportionately high burden, with 2600 estimated premature deaths (40% of the national total) associated with exposure to PM_2.5_, NO₂, and O₃ exposure from these sectors.

Despite these advances, several important gaps remain in the literature regarding the health impacts of air pollution from specific sources. First, most studies have focused on PM_2.5_ and O_3_, with limited attention to NO₂, particularly in terms of chronic exposure. When NO₂ is considered, studies typically assess only acute health effects; yet, long-term exposure to NO_2_ has been related to mortality and other health outcomes (HEI, 2022). Second, previous research has often evaluated the health impacts of interventions on individual pollutants rather than comparably evaluating the health impacts across multiple air pollutants. Third, most existing studies have examined emission reductions at the sectoral level without explicitly considering the benefits of transitioning to cleaner technologies, such as electric vehicles or EPA-certified wood-burning stoves. Additionally, the relatively coarse spatial resolution used in previous air pollution modeling efforts may affect the accuracy of exposure assessments and associated health impact estimations.

To address these gaps, this study employs a chemical transport model to estimate PM_2.5_, NO₂, and O₃ concentrations at a fine resolution and quantifies the associated reductions in mortality across multiple emission sources in the densely populated southern region of Quebec, Canada.

## Materials and methods

### Study area

**Fig. 1:**
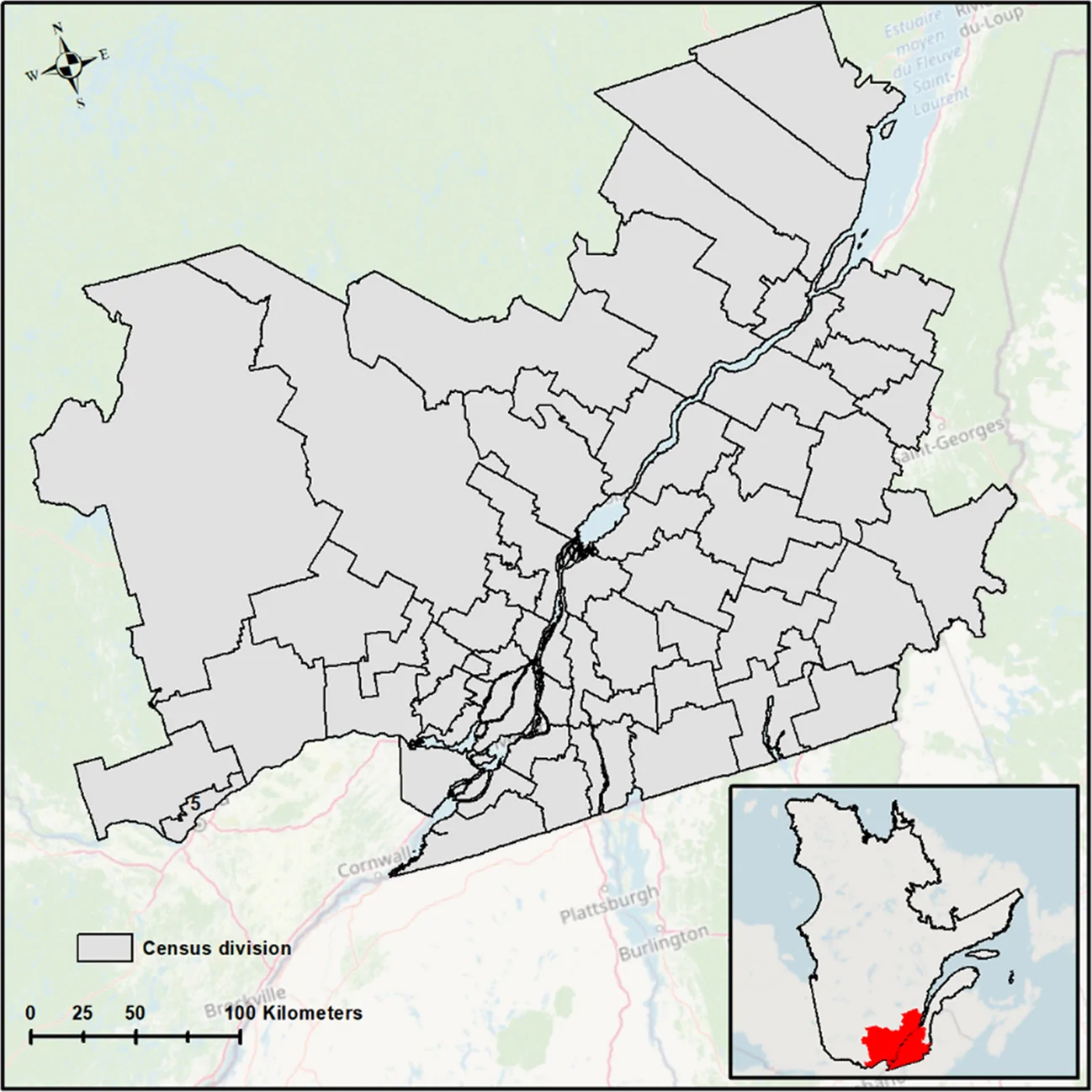
The distribution of census divisions in the study area in Quebec, Canada

Our study area focuses on the densely populated southern region of Quebec, the largest of Canada’s ten provinces. This area encompasses 62 census divisions (CD) and covers approximately 108,000 km^2^, representing just 7.33% of Quebec’s total land area. Yet it accounts for over 87% of the provincial population (Statistics Canada, 2016). Figure 1 displays the study domain and highlights the distribution of these 62 CDs.

### Outline

We developed a series of intervention scenarios based on the 2016 emission database from Canada’s Air Pollutant Emission Inventory (APEI), developed by Environment and Climate Change Canada (ECCC). Using the Polair3D chemical transport model (version *1.11.1*), we estimated the concentrations of PM_2.5_, O₃, and NO₂ at high spatial resolution across the study area for the year 2016. These estimates served as the baseline scenario, against which we also modeled the pollutant concentrations under various intervention scenarios. We then applied the Air Quality Benefits Assessment Tool (AQBAT) to conduct scenario analyses, quantifying the potential health benefits associated with each intervention. We present a comparative analysis to evaluate the effectiveness of these strategies in reducing air pollutant concentrations and mitigating their associated health impacts.

### Intervention scenarios

To identify effective strategies for reducing air pollution and its associated health impacts, we developed four intervention scenarios: (1) replacing all residential wood-burning stoves and fireplaces with EPA-certified alternatives (EPA scenario); (2) eliminating all industrial emissions (IND scenario); (3) fully electrifying the vehicle fleet (EV scenario); and (4) removing emissions from refineries and smelters (RS scenario). While the complete elimination of industrial emissions is not a feasible objective, this scenario was included to provide valuable insights into the sector’s relative contribution to air pollution. These four intervention scenarios were designed to assess the impact of four major emission sources, i.e., residential combustion, industrial processes, transportation, and power generation, on air quality and public health.

### Air pollution modelling

The Polair3D chemical transport model (version *1.11.1*), part of the Polyphemus platform developed at the Centre d’Enseignement et de Recherche en Environnement Atmosphérique (CEREA) in France (Mallet et al., 2007), was employed to map the spatial distribution of the base case PM_2.5_, O₃, and NO₂ at 3 km × 3 km spatial resolution across the study area for the year 2016. The 2016 emission database used as input for the Polair3D model was obtained from Canada’s Air Pollutant Emission Inventory (APEI), developed by Environment and Climate Change Canada (ECCC). To accurately simulate pollutant behavior, we integrated various configurations including the land use data from Copernicus, meteorological data from the Weather Research and Forecasting Model (WRF), and initial and boundary conditions sourced from the Community Atmosphere Model with Chemistry (CAM-Chem). Detailed information on this modelling approach is available in Yamanouchi et al. (2024). The WRF configuration and the Polair3D accuracy assessment are provided in the Supplementary information (Sect. “Introduction”). The model was run seasonally for 4 weeks per season (January for winter, April for spring, July for summer, and October for fall), totaling 16 weeks of simulations. The annual average pollutant concentrations were then calculated as the mean of the four seasonal averages. Following the same procedures, we estimated the PM_2.5_, O₃, and NO₂ concentrations under the four intervention scenarios with scenario emissions.

The Polair3D-modeled concentrations of PM_2.5_, O_3_, and NO_2_, under the base case and the four intervention scenarios, were spatially assigned to each dissemination area (DA). A DA is the smallest standard geographic unit for which all census data, including sociodemographic information, are disseminated in Canada. Figure S2 displays the distribution of DAs and CDs in our study area. To assess exposure at a broader spatial scale, we calculated the population-weighted concentrations at the CD level using the following equation:

1 $${AP}_{CD}=\frac{\sum_{i=1}^{n}{AP}_{DA}^{i} \times {Pop}_{DA}^{i}}{\sum {Pop}_{DA}^{i}}$$

where ${AP}_{CD}$ represents the population-weighted air pollutant concentration for a CD; ${AP}_{DA}^{i}$ and ${Pop}_{DA}^{i}$ are the air pollutant concentration and population of each DA, respectively. *n* is the total number of DAs within the CD. This approach was adopted to compute the PM_2.5_, O_3_, and NO_2_ concentrations at the CD level under the base case and the four scenarios.

Based on Eq. 2, we calculated the population-weighed percentage changes (${\Delta C}_{CD}^{Scn}$) in concentrations of each pollutant for each scenario, in which ${AP}_{CD}^{Base}$ and ${AP}_{CD}^{Scn}$ are the pollutant concentration under base case and scenarios, respectively.

2 $${\Delta C}_{CD}^{Scn}=\frac{{AP}_{CD}^{Base}-{AP}_{CD}^{Scn}}{{AP}_{CD}^{Base}}\times 100$$

The concentration–response functions in AQBAT are not based on the chemical transport models, so we did not directly use the concentrations modelled from Polair3D. Instead, we applied percentage changes (${\Delta C}_{CD}^{Scn}$) obtained from the Polair3D model to the reference concentrations provided by AQBAT. This approach allowed us to derive pollutant concentrations more closely aligned with those typically employed in epidemiological studies and reduce the influence of model biases.

### Modelling PM_2.5_, NO_2_, and O_3_ related mortalities

The AQBAT is a health impact assessment model developed by Health Canada to estimate the health and economic benefits of improvement in air quality. The AQBAT includes relationships between air pollution exposure and a range of health outcomes, including premature mortality, hospital admissions, and respiratory and cardiovascular diseases. The AQBAT applies concentration–response functions from epidemiological studies to quantify health effects, taking into account age, sex, mortality rates, and population distributions across Canada. Detailed information on AQBAT can be found in the Health Canada (2021) report. In this study, AQBAT was used to assess the health impacts of chronic exposure to PM_2.5_ and NO_2_, as well as acute exposure to NO_2_ and O_3_.

AQBAT provides 2016 reference concentrations for PM_2.5_, NO₂, and O₃. While AQBAT includes calculations based on estimates of association for a wide range of health effects, this study focuses specifically on mortality, as it allows for direct comparison across pollutants. Mortality impacts for NO₂ and O₃ are based on acute exposure, whereas those for PM_2.5_ are based on chronic exposure. Additionally, we incorporated mortality risks associated with chronic NO₂ exposure (relative risk (RR) for a 10 μg*/*m^3^ increase, 1.02; 95%CI 1.01–1.04 (Huangfu & Atkinson, 2020)). We added this estimation, which is not included in the AQBAT, as long-term exposure to NO₂, which is a marker for traffic-related air pollution, has been linked to increased mortality (HEI, 2022). The base mortality rate for each CD is provided by AQBAT. Baseline mortality and mortality under the four scenarios were estimated with the respective population-weighted concentrations.

## Results

### Air quality benefits under intervention scenarios

**Fig. 2:**
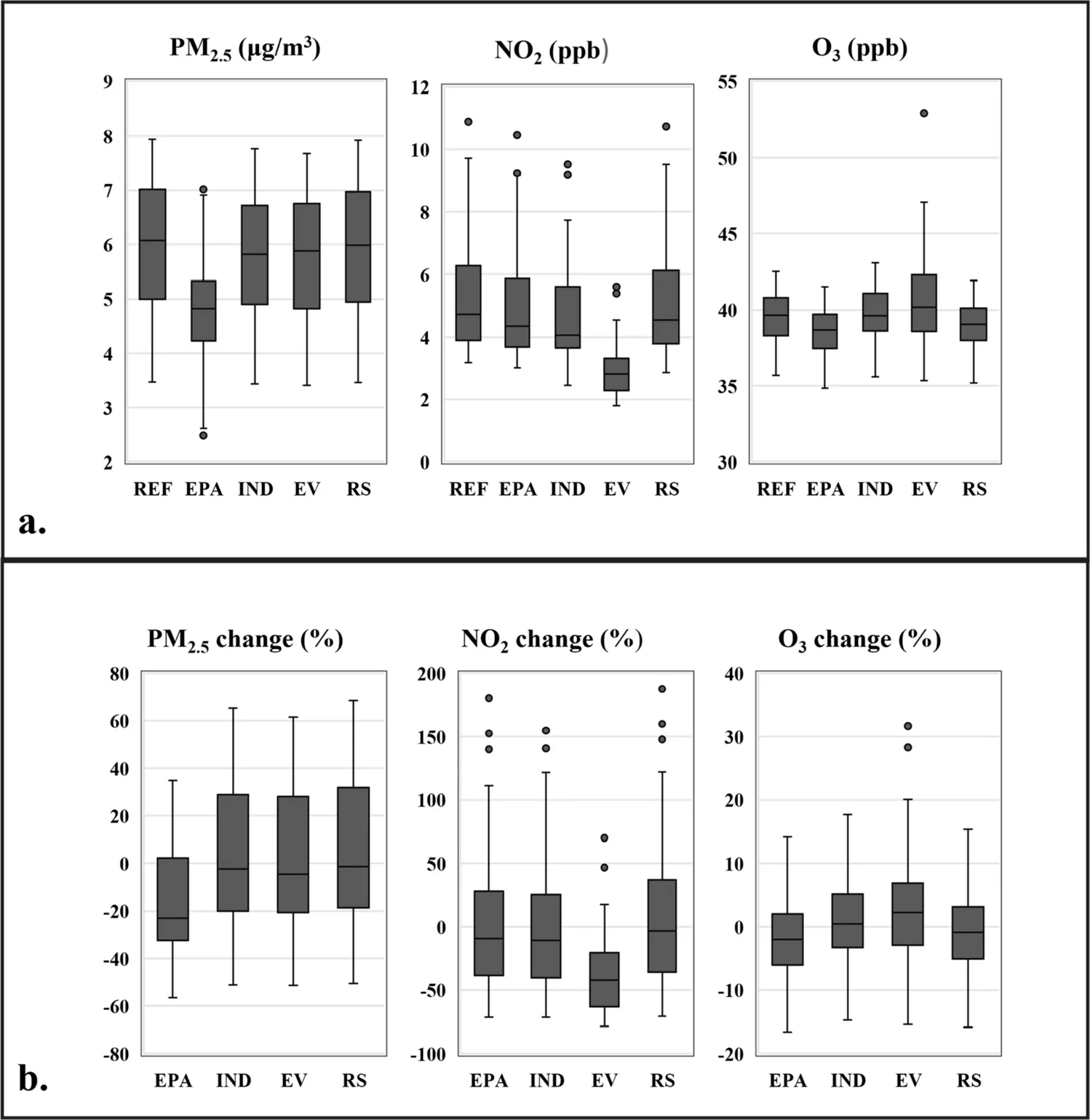
Box plots showing the population-weighted distribution of PM_2.5_, NO_2_, and O_3_ concentrations under the base case (REF) and the four intervention scenarios (**a**) and the corresponding percentage-change distributions (**b**) (EPA for replacing residential wood stoves with EPA certification scenario, IND for eliminating industrial emissions scenario, EV for full vehicle electrification scenario, and RS for removing emissions from refineries and smelters scenario) across the 62 CDs in the study area

**Fig. 3:**
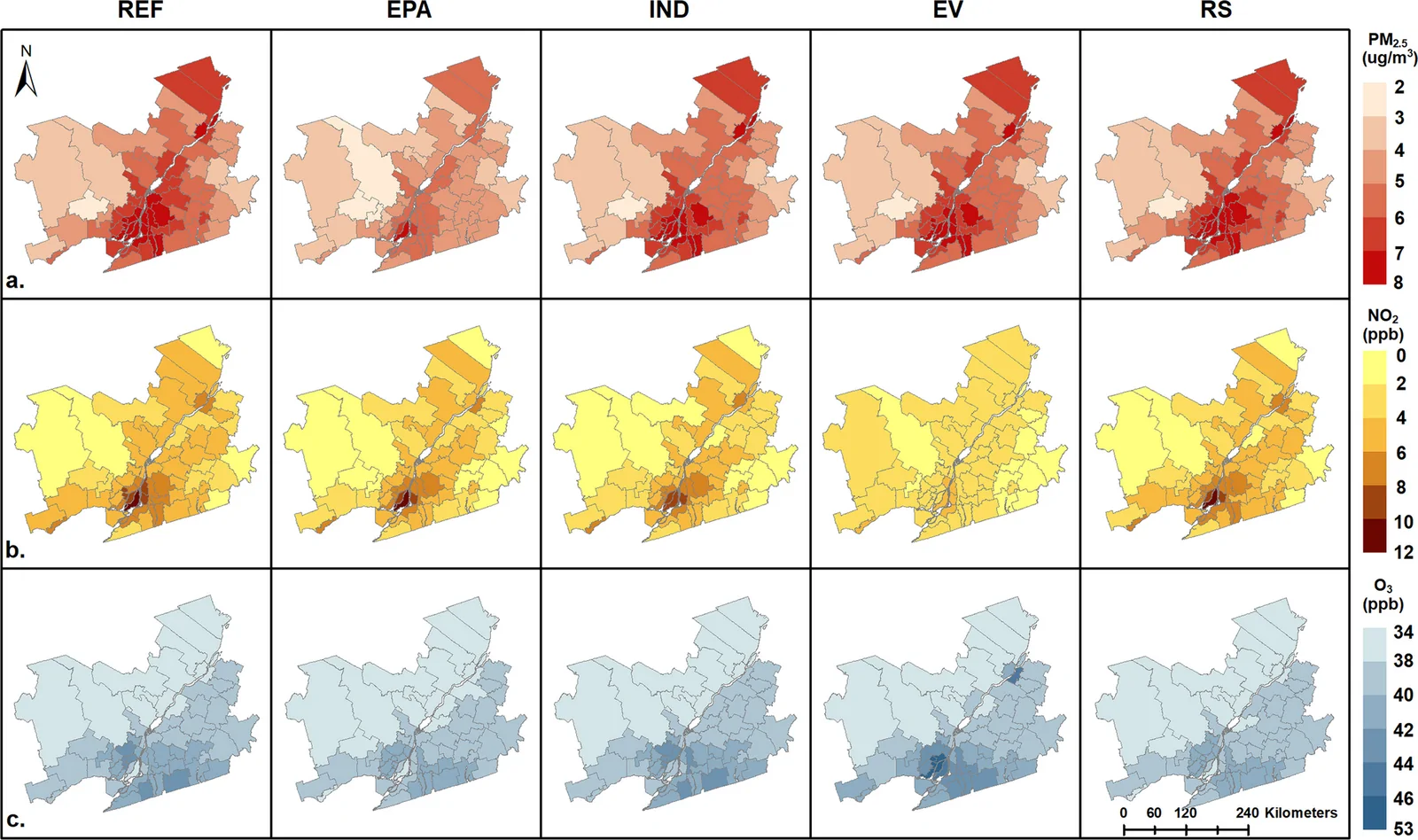
Population-weighted concentrations of PM_2.5_, NO₂, and O₃ at the census division level under the base case (REF), replacing residential wood stoves with EPA certification (EPA), eliminating industrial emissions (IND), full vehicle electrification (EV), and removing emissions from refineries and smelters (RS) scenarios. Pollutant concentrations under the intervention scenarios are estimated by applying percentage changes calculated from the Polair3D model simulations to the reference values from AQBAT tool

Figure 2 illustrates the distributions of population-weighted PM_2.5_, NO₂, and O₃ concentrations under the base case (REF) and four intervention scenarios, and the corresponding percentage change distributions (EPA, IND, EV, and RS) across the 62 CDs in Quebec. Table S2 in the Supplementary information presents descriptive statistics of the concentrations of the three air pollutants under both the base case and the four intervention scenarios, with percentage changes in concentration relative to the base case scenario. Figure 3 depicts their spatial distributions of the three pollutants under the base case and the four scenarios. PM_2.5_, NO₂, and O₃ concentrations remain elevated in high population density areas located in the southern region of Quebec (Fig. 3).

Compared with the base case, all four intervention scenarios lead to reductions in PM_2.5_ and NO₂ concentrations. The EPA scenario is the most effective in reducing PM_2.5_ concentrations, lowering the annual average PM_2.5_ concentration across the 62 CDs by 20%, from 5.90 μg/m^3^ to 4.72 μg/m^3^ (Table S2). In contrast, the PM_2.5_ reductions achieved through eliminating industrial emissions, fully electrifying the vehicle fleet, and removing emissions from refineries and smelters are less pronounced, with annual average concentrations decreasing by less than 4%.

The EV scenario achieves substantial reductions in NO₂ levels. Full electrification of the vehicle fleet leads to the largest reduction, with the annual average NO₂ concentration across the 62 CDs decreasing by 44%, from 5.26 ppb (REF) to 2.94 ppb (EV). The maximum NO₂ concentration also drops by approximately 50%, from 10.88 to 5.63 ppb. The IND and EPA interventions can also reduce NO₂ levels, but to a lesser extent than the EV scenario, with the mean decreasing by 7.2 ~ 9.5%. Removing refineries and smelters (i.e., RS scenario) has the smallest impact, reducing NO₂ levels by only 2.7% (Table S2).

The EV scenarios also resulted in a slight increase in O₃ levels. Under the EV scenario, the annual average maximum daily O₃ concentration across the 62 CDs rises from 39.5 ppb (REF) to 40.6 ppb (EV), with the maximum concentration increasing significantly from 42.5 to 52.9 ppb (Table S2).

### Health benefits under intervention scenarios

**Fig. 4:**
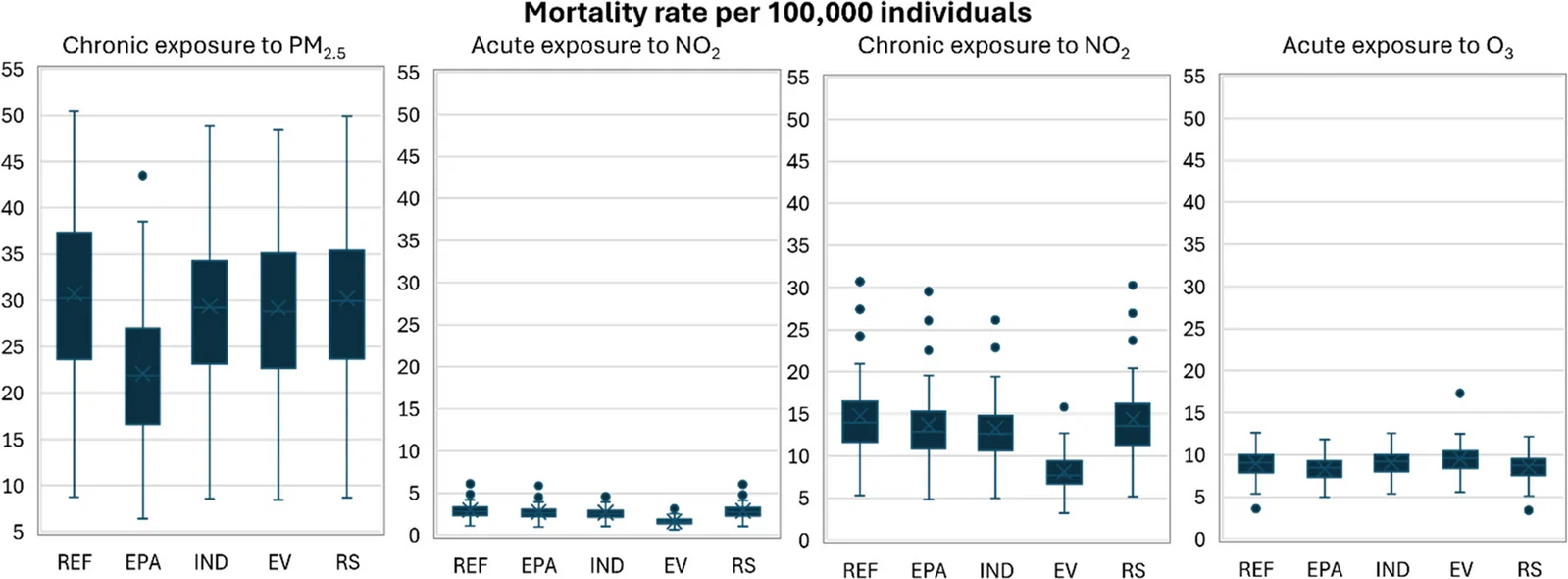
Boxplots showing the annual mortality rates due to chronic exposure to PM_2.5_ and NO_2_ and to acute exposure to NO_2_ and O_3_ across the 62 census divisions across Quebec under the base case (REF) and the replacing residential wood stoves with EPA certification (EPA), eliminating industrial emissions (IND), full vehicle electrification (EV), and removing emissions from refineries and smelters (RS) scenarios

**Fig. 5:**
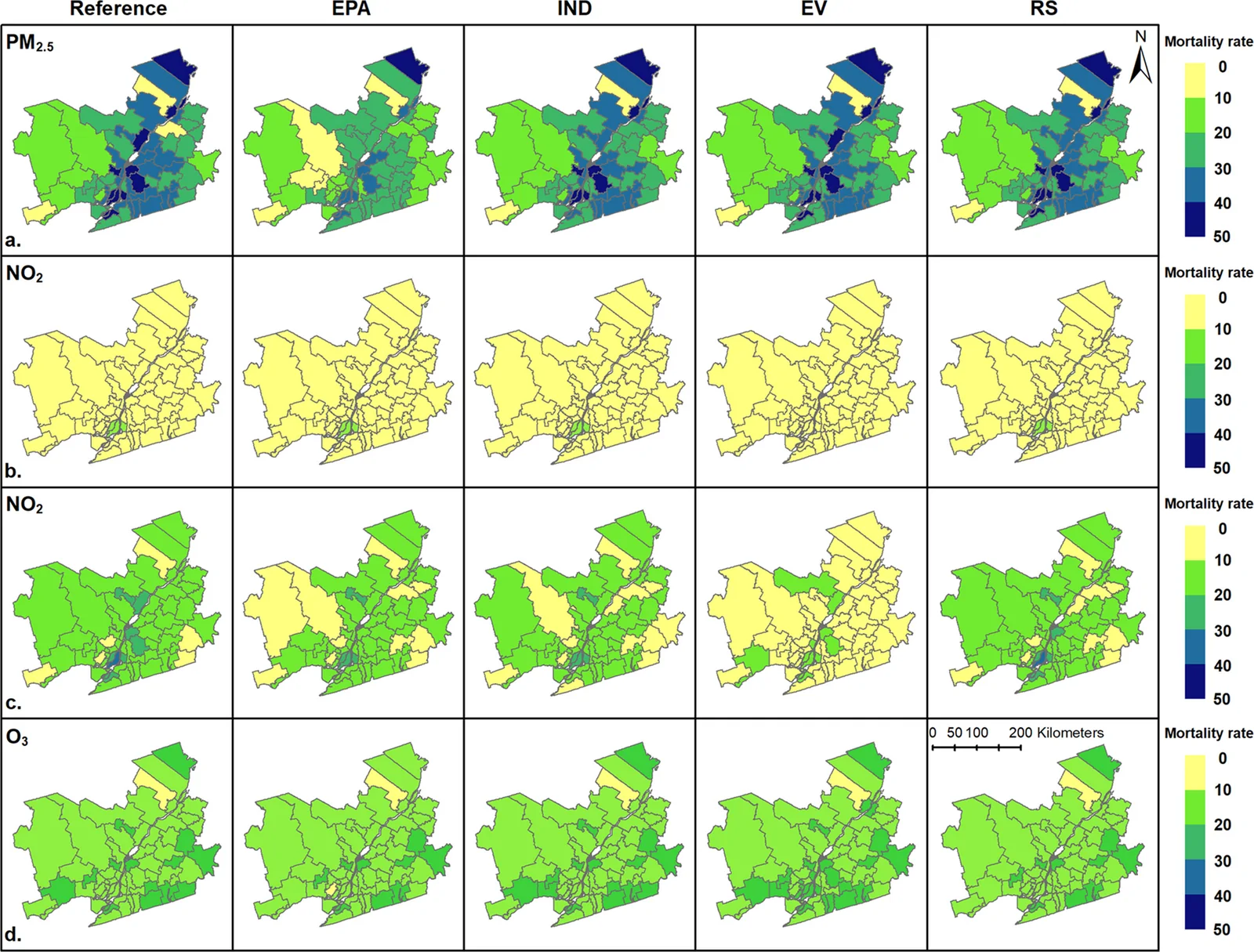
Mortality rates at the census division (CD) level under the base case (REF), replacing residential wood stoves with EPA certification (EPA), eliminating industrial emissions (IND), full vehicle electrification (EV), and removing emissions from refineries and smelters (RS) scenarios. **a** Mortality rate for chronic exposure to PM_2.5_, **b** mortality rate for acute exposure to NO₂, **c** mortality rate for chronic exposure to NO₂, and **d** mortality rate for acute exposure to O₃

Table 1 presents the total number of premature deaths and mortality rates attributed to chronic exposure to PM_2.5_ and NO₂, as well as to acute exposure to NO₂ and O₃. Figure 4 illustrates the distribution of mortality rates linked to these exposures across the 62 CDs in Quebec. Additionally, Fig. 5 maps the spatial distribution of mortality rates under the base case and the four intervention scenarios, while Table S3 provides a statistical summary of mortality rates for each pollutant. Health benefits observed are coherent with pollutant reductions. Overall, all intervention scenarios yield health benefits by reducing premature deaths and death rates. However, the IND and EV interventions result in a slight increase in premature deaths and death rates due to acute exposure to O₃.

**Table 1:** Total number of annual deaths and mean mortality rates for the 62 census divisions studied under the base case and the four intervention scenarios and the percentage change between base case and each of the scenarios

	**Number of deaths (change %)**				
	REF	EPA	IND	EV	RS
Chronic exposure to PM_2.5_	2732	2084 (−23.72%)	2633 (−3.62%)	2590 (−5.20%)	2711 (−0.77%)
Acute exposure to NO_2_	309	292 (−5.50%)	280 (−9.39%)	158 (−48.87%)	303 (−1.94%)
Chronic exposure to NO_2_	1547	1461 (−5.56%)	1400 (−9.50%)	795 (−48.61%)	1517 (−1.94%)
Acute exposure to O_3_	581	543 (−6.54%)	620 (6.71%)	792 (36.32%)	559 (−3.79%)
	**Mortality rates (change %)**				
Chronic exposure to PM_2.5_	30.71	22.05 (−28.20%)	29.35 (−4.43%)	29.18 (−4.98%)	30.20 (−1.66%)
Acute exposure to NO_2_	2.96	2.74 (−7.43%)	2.66 (−10.14%)	1.62 (−45.27%)	2.88 (−2.70%)
Chronic exposure to NO_2_	14.75	13.67 (−7.32%)	13.28 (−9.97%)	8.13 (−44.88%)	14.36 (−2.64%)
Acute exposure to O_3_	8.94	8.32 (−6.94%)	9.05 (1.23%)	9.55 (6.82%)	8.59 (−3.91%)

*REF* represents for the base case, *EPA* for replacing residential wood stoves with EPA certification scenario, *IND* for eliminating industrial emissions scenario, *EV* for full vehicle electrification scenario, and *RS* for removing emissions from refineries and smelters scenario

Among the four intervention scenarios, the EPA scenario offers the greatest health benefits associated with long-term exposure to PM_2.5_. Compared with the base case (REF), replacing residential wood stoves with EPA-certified stoves reduces mortality from chronic PM_2.5_ exposure by 23.72%, preventing 648 estimated premature deaths (Table 1). Additionally, the average mortality rate under the EPA scenario decreased by 28.2% (Fig. 4), with the most substantial reductions observed in the Greater Montreal Region (Fig. 5). While the EV and IND interventions also contribute to reducing PM_2.5_-attributable deaths, their impact is notably smaller, with reductions of only 5.20% and 3.62% premature deaths, respectively.

Full vehicle electrification is the most effective intervention for reducing mortality and mortality rates associated with both acute and chronic exposure to NO₂. Under the EV scenario, mortality due to acute and chronic NO₂ exposure decreases by nearly 50%, preventing 151 and 752 estimated premature deaths, respectively. The average mortality rates associated with acute and chronic NO₂ exposure decline by 45.27% and 42.69%, respectively (Fig. 4). These reductions are observed across all 62 census divisions (CDs) under the EV intervention scenario (Fig. 5). While eliminating industrial emissions (IND) also reduces NO₂-related premature deaths, its impact is considerably smaller, leading to only a 10% decrease for both acute and chronic exposure. The EPA stove intervention provides additional benefits in reducing NO₂-related mortality; however, its effect is approximately half as effective as that observed under the IND scenario.

The EPA stove and RS (closure of refineries and smelters) interventions provide additional health benefits by reducing premature deaths associated with acute exposure to O₃. Specifically, the number of premature deaths decreases by 6.54% and 3.97% under the EPA and RS scenarios, respectively. In contrast, the IND and EV scenarios result in a slight increase in O₃-related premature deaths (Table 1). However, the overall impact of all interventions on mortality rates due to acute O₃ exposure is minimal, with changes of less than 1% across all four scenarios (Fig. 4).

## Discussion

The findings of this study highlight that considering mortality effects of chronic air pollutant exposure, replacing all wood stoves with EPA-certified stoves yields comparable benefits to electrifying all road vehicles. Although these two intervention scenarios are associated with similar death reductions, their mechanisms of action differ significantly. While the EPA stove scenario primarily reduces mortality associated with chronic PM_2.5_ exposure, the EV scenario is most effective in mitigating mortality from chronic NO₂ exposure. It is worth noting that electric vehicles eliminate NO₂ emissions; they still contribute to PM_2.5_ emissions through non-exhaust sources such as brake wear, tire wear, and road dust resuspension (Timmers & Achten, 2016), and these were not removed from our EV scenario.

### Comparison with previous studies

Our results can be compared to results from a recent report from Health Canada (2023). Health Canada (2023) modelled pollutant levels from 21 sectors with the GEM-MACH system at a 10 km × 10 km resolution, then applied the AQBAT to evaluate the associated mortality burden across Canadian provinces. The residential, industrial, and transportation sectors were identified as the primary contributors to mortality linked to air pollutants, collectively accounting for 6500 premature deaths. In Quebec, 2300 estimated premature deaths were attributed to chronic PM_2.5_ exposure and acute NO₂ and O₃ exposure, representing 40% of the total premature deaths in Canada. Our study yields comparable estimates, with 2732 premature deaths attributed to chronic PM_2.5_ exposure, 309 deaths due to acute NO₂ exposure, and 581 deaths due to acute O₃ exposure in Quebec.

Health Canada (2023) identified residential wood burning as the dominant contributor to premature deaths, particularly due to PM_2.5_ exposure, which was responsible for 68% (1400 deaths) of chronic PM_2.5_-related mortality. Our analysis similarly found residential wood burning as the primary source of PM_2.5_-related deaths. We estimated that replacing conventional residential wood stoves with EPA-certified models could reduce premature deaths by 682 from chronic PM_2.5_ exposure, accounting for 74% of the total reduction achieved by eliminating emissions from residential wood burning, industrial activities, and on-road traffic. However, our estimate of PM_2.5_-related mortality from residential wood burning (682 deaths) is lower than Health Canada’s estimate (1400 deaths). This difference stems from the fact that our analysis does not encompass all of Quebec. More importantly, while EPA-certified wood stoves produce fewer emissions than conventional models, they still contribute to PM_2.5_ pollution, which likely explains the lower estimated reduction in premature deaths.

However, our analysis suggests that the impact of on-road transportation is more critical than previously estimated, such as by Health Canada (2023). Traffic emissions are a dominant source of NO₂, and while past studies have primarily focused on the acute health effects of NO₂ exposure, emerging evidence suggests that chronic exposure to traffic-related air pollution poses significant health risks (Chen et al., 2024; Huang et al., 2021). Unlike previous assessments, our study considered both acute and chronic NO₂ exposure. We found that fully electrifying vehicles (eliminating on-road transportation emissions) could prevent 151 premature deaths from acute NO₂ exposure and 752 premature deaths from chronic NO₂ exposure. Notably, the reduction in chronic NO₂-related mortality is very similar to the estimated reduction in chronic PM_2.5_-related mortality from residential wood burning emission control, underscoring the critical role of traffic emissions in air pollution-related health impacts.

In contrast to the Health Canada (2023) report, which found that removing emissions from on-road transportation would reduce 28 premature deaths from acute O₃ exposure, our analysis yielded the opposite conclusion: we observed an increase in premature deaths associated with acute O₃ exposure following the removal of on-road transportation emissions. This discrepancy can be due to differences in the spatial resolution of the chemical transport models used. The increase that we noted is coherent with other studies (Blanchard & Tanenbaum, 2003; Wang et al., 2023; Wu et al., 2022) and is likely due to the complex chemistry of ground-level O₃ formation. Unlike primary pollutants, O₃ is not emitted directly but forms when nitrogen oxides (NOₓ) and volatile organic compounds react in sunlight. On-road transportation sources release large amounts of NO_x_, which can create and destroy O₃. In areas with high NOₓ levels, such as cities with heavy traffic, NOₓ reacts with O₃ and removes it from the air (Atkinson, 2000). As a result, reducing NO_x_ emissions can lead to an increase in O_3_ concentrations, potentially offsetting expected health benefits.

Overall, the comparison between our findings and those of Health Canada highlights the importance of methodological choices such as the use of chronic effects risk functions, spatial resolution, and pollutant interactions in assessing air pollution-related health impacts.

### Advantages of this study

This study offers several key advantages over previous assessments. First, the spatial resolution of the chemical transport model used enables higher-resolution estimates of PM_2.5_, NO₂, and O₃ concentrations, enhancing the accuracy of health impact evaluations. The finer spatial resolution (3 km × 3 km) provides a more precise representation of pollutant distribution, particularly in urban areas where air quality can vary significantly over short distances.

Second, our study incorporates multiple intervention scenarios, allowing for a comprehensive comparison of health benefits across different air pollution sources. This approach provides a clearer understanding of which mitigation strategies yield the greatest health benefits while considering cost-effectiveness. For example, our results indicate that replacing residential wood stoves with EPA-certified models and full vehicle electrification achieve a similar decrease in mortality.

Third, while studies on air pollution mitigation often focus on reducing emissions from residential and industrial sources, limited research has assessed the health benefits of full vehicle electrification. This issue is particularly relevant in Quebec, which leads all Canadian provinces in electric vehicle adoption. As of the third quarter of 2024, Quebec had 40,763 registered zero-emission vehicles, accounting for 22.7% of all new vehicle registrations in the province (Statistics Canada, 2024). Given that Quebec’s high adoption rate is largely driven by substantial government incentives, our findings highlight the critical role of vehicle electrification in reducing air pollution-related mortality, informing future transportation policies and investment decisions.

Finally, unlike many previous studies that primarily focus on acute NO₂ exposure, our analysis separately quantifies the health impacts of both acute and chronic NO₂ exposure. Chronic NO₂ exposure is associated with a significantly higher number of premature deaths than acute exposure, reinforcing its long-term health risks. By incorporating chronic NO₂ mortality, our study underscores the need for policies that prioritize sustained reductions in traffic-related air pollution, rather than focusing solely on short-term interventions.

### Limitations

This study provides valuable insights into the health impacts of air pollution and the benefits of emission reduction strategies, while several limitations should be acknowledged.

First, our analysis relies on CRFs derived from observational studies. This approach assumes that CRFs can accurately predict the causal relationships between changes in air pollution and health outcomes due to interventions. However, CRFs are estimated from population-level data and, as a result, they may reflect correlations influenced by unmeasured factors such as socioeconomic status and co-pollutants, rather than true causal effects (Cox, 2017). This potential mismatch brings uncertainty in health impact estimates. Therefore, further research is needed to reinforce our results.

Second, our analysis primarily focused on mortality outcomes, while other critical health effects, such as hospital admissions, respiratory illnesses, and cardiovascular diseases, were not comprehensively evaluated. Future research incorporating these non-fatal health outcomes would provide a more complete picture of air pollution’s public health burden. Also, sulfur dioxide (SO₂), a well-known industrial pollutant, was excluded from our analysis due to the limited availability of exposure data in the AQBAT model, which only encompasses six census divisions. Nonetheless, the health impacts of SO₂ are primarily associated with acute exposures and are unlikely to substantially influence our conclusions.

Third, while air pollutants were modelled with the Polair3D chemical transport model at high spatial resolution, pollutant concentration estimates are subject to inherent uncertainties and potential biases (Yamanouchi et al., 2024). To mitigate these limitations, we did not use absolute Polair3D-modeled concentrations to estimate health impacts. Instead, we applied percentage changes in pollutant levels from Polair3D to the AQBAT tool’s baseline concentrations. Although this approach helps reduce systematic biases, percent changes could be inaccurate, especially when nonlinear responses that are poorly represented by model processes exist (e.g., for ozone formation). Applying percent changes may also smooth out local differences, which could lead to over- or underestimating health benefits in specific areas.

Additionally, the health effects of PM_2.5_ exposure from wood combustion may be overestimated in our study. The AQBAT model applies chronic exposure estimates, assuming long-term and recurring exposure over several months or years. However, PM_2.5_ emissions from wood combustion are more episodic and may be more accurately linked to acute rather than chronic exposure. Since acute exposures typically have lower associated health risks than chronic exposures, our findings may overstate the health benefits of reducing residential wood combustion. Further research is needed to refine these estimates and differentiate between the health effects of short- and long-term exposure to PM_2.5_ from wood burning.

Furthermore, the AQBAT tool does not account for health impacts associated with volatile organic compounds (VOCs), which play a role in the formation of secondary pollutants, including O₃ and PM₂.₅. Although our study indirectly considers VOC effects through their influence on these pollutants, a more explicit assessment of VOC-related health impacts remains an important area for future research. Similarly, the AQBAT model does not estimate health risks from specific toxic pollutants such as heavy metals, due to limited available data on their exposure–response relationships in non-occupational settings. Given the growing evidence linking airborne metal exposure to adverse health outcomes, further research is needed to better understand their health effects, namely in industrial towns.

Finally, future work should focus on improving air pollution modeling accuracy, incorporating additional environmental factors such as climate change, and expanding the range of health outcomes assessed. By addressing these limitations, future studies can enhance our understanding of air pollution’s full health burden and help refine strategies for mitigating its effects.

## Contributions to knowledge

What does this study add to existing knowledge?

- This study provides a comparative, multi-pollutant assessment (PM_2.5_, NO₂, O₃) of four interventions on health impacts (replacing residential wood stoves with EPA-certified models, eliminating industrial emissions, full vehicle electrification, and removing emissions from refineries and smelters) at fine spatial resolution (3 × 3 km) across 62 census divisions in southern Quebec, Canada.
- It explicitly quantifies mortality from chronic NO₂ exposure, addressing a gap in prior work that focused primarily on acute NO₂.
- It identifies full vehicle electrification and EPA-certified wood-stove replacement as the most effective interventions, producing the greatest reductions in NO₂- and PM_2.5_-attributable mortality, respectively.

What are the key implications for public health interventions, practice, or policy?

- EPA-certified wood stove replacement and full vehicle electrification have to be the leading policy priorities for mitigating mortality from PM_2.5_ and NO₂ exposure.
- Chronic NO₂ exposure needs to be considered in health and policy analyses because relying only on acute NO₂ misses important traffic-related risks.
- The high-burden geographic areas need to be identified to maximize health benefits.

## Supplementary Information

Below is the link to the electronic supplementary material.ESM1(DOCX 791 KB)

## Data Availability

The air pollution concentration data used in this study were derived from simulations using the Polair3D chemical transport model and from reference values provided by the Air Quality Benefits Assessment Tool (AQBAT) by Health Canada. Mortality data were obtained from the AQBAT database. All data used are publicly available or available upon reasonable request from the corresponding author.

## Code availability

The Polair3D model is open source and available through the Polyphemus modeling platform. Custom R scripts used to process data and perform the analysis are available from the corresponding author upon reasonable request.
