# Supplementary material for: Clearing the air: Which pollution source matters most for health?
Source: Can J Public Health. 2025 Dec 9;117(4):703–14. doi: 10.17269/s41997-025-01142-1 (PMC13534371; doi:10.17269/s41997-025-01142-1)
Supplement: Supplementary file 1 [file 41997_2025_1142_MOESM1_ESM.docx]

**Clearing the air: which pollution source matters most for health?**

**Supplementing Information**

**1. Modelling framework, configuration, and seasonal design**

The Polair3D chemical transport model was employed to estimate the PM_2.5_, NO₂, and O₃ across southern Quebec following Yamanouchi *et al.* (2024). Model performance was assessed against multiple reference datasets, including ground measurements from the National Air Pollution Surveillance (NAPS) network, land-use-regression NO₂ (Canadian Urban Environmental Health Research Consortium), and PM_2.5_ from the Atmospheric Composition Analysis Group. **Table S1** summarizes evaluation metrics (R, MRD, MSE [µg/m³], MB [µg/m³], NMB, and N), providing an overview of model performance for each pollutant.

**Table S1**. Model evaluation metrics for Polair3D-modelled PM_2.5_, NO₂, and O₃ compared with reference datasets.

|  | **N** | **R** | **MRD** | **MSE** | **MB** | **NMB** |
| --- | --- | --- | --- | --- | --- | --- |
| **PM_2.5_** | 123 | 0.45 | -3.90 | 22.1 | 1.8 | 26.3 |
| **NO_2_** | 75 | 0.70 | 33.5 | 214 | 11.5 | 78 |
| **O_3_** | 144 | 0.85 | 21.4 | 466 | 19.5 | 27.3 |

Notes: N for the number of monitoring sites; R for correlation coefficient, MRD for mean relative difference, MSE for mean squared error, MB for mean bias, NMB for normalized mean bias.

Meteorology was generated with WRF using four two-way nested domains at 27, 9, 3, and 1 *km* horizontal resolution and 42 vertical levels (surface to 100 *hPa*). Initial and boundary conditions were from NARR (32 *km*, 30 levels). We performed 30-h forecasts initialized daily at 00:00 UTC with a 6-h spin-up; the first 6 h were discarded and replaced with overlapping forecasts. Grid nudging of wind, temperature, and humidity was applied above the planetary boundary layer in the outermost domain. Physics parameterizations included the Purdue–Lin microphysics scheme, RRTMG radiation, Mellor–Yamada–Janjic PBL, Grell–Devenyi cumulus (domains 1–2 only), the Unified Noah land-surface model, and a three-category urban canopy model. Additional configuration details are provided in Yamanouchi *et al.* (2024).

**Figure S1.** Monthly mean temperatures at Montréal–Trudeau International Airport for 2016, with seasonal (Winter: Dec–Feb, Spring: Mar–May, Summer: Jun–Aug, Fall: Sep–Nov) and annual averages shown alongside the 1991–2020 climate normals (Available from: <https://climate.weather.gc.ca/climate_normals/results_1991_2020_e.html?searchType=stnName_1991&txtStationName_1991=montreal&searchMethod=contains&txtCentralLatMin=0&txtCentralLatSec=0&txtCentralLongMin=0&txtCentralLongSec=0&stnID=123000000&dispBack=1> ).

To ensure seasonal representativeness while maintaining computational feasibility, we simulated four representative weeks: January (winter), April (spring), July (summer), and October (fall). **Figure S1** shows monthly mean temperatures at Montréal–Trudeau International Airport for 2016 alongside seasonal (Winter: December to February; Spring: March to May; Summer: June to August; Fall: September to November) and annual averages, as well as 1991–2020 climate normals. The selected months closely match their respective seasonal means, capturing essential seasonal variability in pollutant levels while avoiding the prohibitive cost of simulating all twelve months. This seasonal-week design is consistent with practices in the climate-modelling literature (Montgomery *et al*., 2023; Camilleri *et al.*, 2023; Minet *et al.*, 2021).

**Table S2.** Descriptive statistics of annual average daily PM_2.5_, annual mean NO_2_, and annual average maximum daily O_3_ in census divisions under base case and various scenarios. Values are reported as absolute concentrations with percentage changes relative to reference concentrations (Note: PM_2.5_, NO_2_, and O_3_ were derived by applying the Polair3D-modelled percentage change to the AQBAT reference concentrations).

|  | | REF | EPA  (Change %) | IND  (Change %) | EV  (Change %) | RS  (Change %) |
| --- | --- | --- | --- | --- | --- | --- |
| PM_2.5_  (μg/m^3^) | Minimum | 3.47 | 2.48 (-28.53%) | 3.44 (-28.53 %) | 3.41 (-28.53%) | 3.46 (-28.53%) |
|  | Q1^*^ | 5.05 | 4.25 (-15.84%) | 4.95 (-15.84%) | 4.88 (-15.84%) | 5.00 (-15.84%) |
|  | Median | 6.08 | 4.82 (-20.72%) | 5.82 (-20.72%) | 5.88 (-20.72%) | 5.98 (-20.72%) |
|  | Mean | 5.9 | 4.72 (-20.00 %) | 5.74 (-20.00%) | 5.70 (-20.00%) | 5.85 (-20.00%) |
|  | Q3^#^ | 7 | 5.31 (-24.14%) | 6.66 (-24.14%) | 6.71 (-24.14%) | 6.96 (-24.14%) |
|  | Maximum | 7.94 | 7.01 (-11.71%) | 7.77 (-11.71%) | 7.67 (-11.71%) | 7.92 (-11.71%) |
|  |  |  |  |  |  |  |
| NO_2_  (ppb) | Minimum | 3.17 | 3.01 (-5.05%) | 2.44 (-5.05%) | 1.80 (-5.05%) | 2.86 (-5.05%) |
|  | Q1 | 3.92 | 3.68 (-6.12%) | 3.66 (-6.12%) | 2.30 (-6.12%) | 3.82 (-6.12%) |
|  | Median | 4.72 | 4.35 (-7.84%) | 4.05 (-7.84%) | 2.81 (-7.84%) | 4.55 (-7.89%) |
|  | Mean | 5.26 | 4.88 (-7.22%) | 4.76 (-7.22%) | 2.94 (-7.22%) | 5.12 (-7.22%) |
|  | Q3 | 6.21 | 5.80 (-6.60%) | 5.54 (-6.60%) | 3.32 (-6.60%) | 6.06 (-6.60%) |
|  | Maximum | 10.88 | 10.46 (-3.86%) | 9.52 (-3.86%) | 5.63 (-3.86%) | 10.73 (-3.86%) |
|  |  |  |  |  |  |  |
| O_3_  (ppb) | Minimum | 35.7 | 34.90 (-2.24%) | 35.60 (-2.24%) | 35.40 (-2.24%) | 35.20 (-2.24%) |
|  | Q1 | 38.4 | 37.60 (-2.08%) | 38.70 (-2.08%) | 38.60 (-2.08%) | 38.00 (-2.08%) |
|  | Median | 39.7 | 38.70 (-2.52%) | 39.60 (-2.52%) | 40.20 (-2.52%) | 39.10 (-2.52%) |
|  | Mean | 39.5 | 38.60 (-2.28%) | 39.70 (-2.28%) | 40.60 (-2.28%) | 39.00 (-2.28%) |
|  | Q3 | 40.7 | 39.70 (-2.46 %) | 41.00 (-2.46%) | 42.30 (-2.46%) | 40.00 (-2.46%) |
|  | Maximum | 42.5 | 41.50 (-2.35%) | 43.10 (-2.35%) | 52.90 (-2.35%) | 41.90 (-2.35%) |

Note: Q1^*^ and Q3^#^ stand for the 25% and 75% quantiles. REF represents for the base case, EPA for replacing residential wood stoves with EPA certification scenario, IND for eliminating industrial emissions scenario, EV for full vehicle electrification scenario, and RS for removing emissions from refineries and smelters scenario.

**Table S3.** Descriptive statistics of mortality rates per 100,000 persons due to exposure to PM_2.5_, NO_2_, and O_3_ in census divisions under base case and various scenarios.

|  |  | **REF** | **EPA** | **IND** | **EV** | **RS** |
| --- | --- | --- | --- | --- | --- | --- |
| Chronic exposure to PM_2.5_ | Minimum | 8.70 | 6.40 | 8.50 | 8.50 | 8.70 |
|  | Q1 | 24.00 | 16.73 | 23.40 | 22.88 | 24.03 |
|  | Median | 30.20 | 21.90 | 29.20 | 28.75 | 29.90 |
|  | Mean | 30.71 | 22.05 | 29.35 | 29.18 | 30.20 |
|  | Q3 | 35.80 | 26.70 | 33.85 | 34.25 | 34.58 |
|  | Maximum | 50.50 | 43.50 | 48.90 | 48.50 | 49.90 |
|  |  |  |  |  |  |  |
| Acute exposure to NO_2_ | Minimum | 1.10 | 1.00 | 1.00 | 0.60 | 1.00 |
|  | Q1 | 2.33 | 2.20 | 2.20 | 1.40 | 2.30 |
|  | Median | 2.80 | 2.60 | 2.50 | 1.50 | 2.70 |
|  | Mean | 2.96 | 2.74 | 2.66 | 1.62 | 2.88 |
|  | Q3 | 3.28 | 3.08 | 2.90 | 1.88 | 3.20 |
|  | Maximum | 6.10 | 5.90 | 5.40 | 3.10 | 6.00 |
|  |  |  |  |  |  |  |
| Chronic exposure to NO_2_ | Minimum | 5.30 | 4.80 | 5.00 | 3.20 | 5.20 |
|  | Q1 | 11.78 | 11.03 | 10.88 | 6.73 | 11.43 |
|  | Median | 13.95 | 12.85 | 12.65 | 7.65 | 13.60 |
|  | Mean | 14.75 | 13.67 | 13.28 | 8.13 | 14.36 |
|  | Q3 | 16.28 | 15.18 | 14.68 | 9.33 | 15.95 |
|  | Maximum | 30.70 | 29.50 | 26.90 | 15.90 | 30.30 |
|  |  |  |  |  |  |  |
| Acute exposure to O_3_ | Minimum | 3.60 | 3.30 | 3.60 | 3.40 | 3.40 |
|  | Q1 | 7.93 | 7.40 | 8.00 | 8.48 | 7.63 |
|  | Median | 9.05 | 8.50 | 9.20 | 9.60 | 8.75 |
|  | Mean | 8.94 | 8.32 | 9.05 | 9.55 | 8.59 |
|  | Q3 | 9.98 | 9.28 | 10.00 | 10.30 | 9.58 |
|  | Maximum | 12.60 | 11.80 | 12.50 | 17.30 | 12.20 |

Note: REF represents for the base case, EPA for replacing residential wood stoves with EPA certification scenario, IND for eliminating industrial emissions scenario, EV for full vehicle electrification scenario, and RS for removing emissions from refineries and smelters scenario.

**Figure S2**. The distributions of census divisions and dissemination areas in the study area.

**References**

Yamanouchi, S., Gamage, S. M., Torbatian, S., Zalzal, J., Minet, L., Smargiassi, A., ... & Hatzopoulou, M. (2024). Validation and analysis of the Polair3D v1. 11 chemical transport model over Quebec. *Geoscientific model development*, 17(9), 3579-3597.

NAPS. National Air Pollution Surveillance (NAPS) Program. Available from : <https://open.canada.ca/data/en/dataset/1b36a356-defd-4813-acea-47bc3abd859b>, last accessed September 29, 2025.

Hystad, P., Setton, E., Cervantes, A., Poplawski, K., Deschenes, S., Brauer, M., ... & Demers, P. (2011). Creating national air pollution models for population exposure assessment in Canada. *Environmental health perspectives*, 119(8), 1123-1129.

van Donkelaar, A., Hammer, M. S., Bindle, L., Brauer, M., Brook, J. R., Garay, M. J., ... & Martin, R. V. (2021). Monthly global estimates of fine particulate matter and their uncertainty. *Environmental science & technology*, 55(22), 15287-15300.

Montgomery, A., Schnell, J. L., Adelman, Z., Janssen, M., & Horton, D. E. (2023). Simulation of neighborhood‐scale air quality with two‐way coupled WRF‐CMAQ over Southern Lake Michigan‐Chicago Region. *Journal of geophysical research: atmospheres*, 128(6), e2022JD037942.

Camilleri, S. F., Montgomery, A., Visa, M. A., Schnell, J. L., Adelman, Z. E., Janssen, M., ... & Horton, D. E. (2023). Air quality, health and equity implications of electrifying heavy-duty vehicles. *Nature sustainability*, 6(12), 1643-1653.

Minet, L., Wang, A., & Hatzopoulou, M. (2021). Health and climate incentives for the deployment of cleaner on-road vehicle technologies. *Environmental science & technology*, 55(10), 6602-6612.
